# Control measures to prevent the increase of paratuberculosis prevalence in dairy cattle herds: an individual-based modelling approach

**DOI:** 10.1186/s13567-018-0557-3

**Published:** 2018-07-13

**Authors:** Guillaume Camanes, Alain Joly, Christine Fourichon, Racem Ben Romdhane, Pauline Ezanno

**Affiliations:** 1 Groupement de Défense Sanitaire de Bretagne, 56019 Vannes, France; 2 BIOEPAR, INRA, Oniris, Université Bretagne Loire, 44307 Nantes, France

## Abstract

Paratuberculosis, a gastrointestinal disease caused by *Mycobacterium avium* subsp. *paratuberculosis* (Map), can lead to severe economic losses in dairy cattle farms. Current measures are aimed at controlling prevalence in infected herds, but are not fully effective. Our objective was to determine the most effective control measures to prevent an increase in adult prevalence in infected herds. We developed a new individual-based model coupling population and infection dynamics. Animals are characterized by their age (6 groups) and health state (6 states). The model accounted for all transmission routes and two control measures used in the field, namely reduced calf exposure to adult faeces and test-and-cull. We defined three herd statuses (low, moderate, and high) based on realistic prevalence ranges observed in French dairy cattle herds. We showed that the most relevant control measures depend on prevalence. Calf management and test-and-cull both were required to maximize the probability of stabilizing herd status. A reduced calf exposure was confirmed to be the most influential measure, followed by test frequency and the proportion of infected animals that were detected and culled. Culling of detected high shedders could be delayed for up to 3 months without impacting prevalence. Management of low prevalence herds is a priority since the probability of status stabilization is high after implementing prioritized measures. On the contrary, an increase in prevalence was particularly difficult to prevent in moderate prevalence herds, and was only feasible in high prevalence herds if the level of control was high.

**Electronic supplementary material:**

The online version of this article (10.1186/s13567-018-0557-3) contains supplementary material, which is available to authorized users.

## Introduction

Paratuberculosis, a gastrointestinal infection commonly reported in cattle, is an incurable disease caused by *Mycobacterium avium* subsp. *paratuberculosis* (Map), a pathogen highly resistant in the environment [1, 2]. Multiple transmission routes are involved in the infection process, mainly during the first months of life [3, 4]. The first is vertical (in utero) from the dam to calf [5]. The second is horizontal, resulting from the ingestion of contaminated milk or colostrum, or after the ingestion of contaminated faeces from adults or from other calves [3, 6, 7]. Paratuberculosis presents a slow evolution in various infection stages, which display heterogeneous shedding patterns [8–10]. In late infection, visible clinical signs may occur, such as profuse diarrhoea, emaciation, decreased milk production, and significant health impairment that can lead to early animal death [11].

Due to the direct effects of infection and the possible restrictions in trading live animals, paratuberculosis causes considerable economic losses [12]. Paratuberculosis is present worldwide and more than 50% of herds can be infected in countries with a significant dairy industry [13]. Within-herd prevalence is heterogeneous, ranging from 2.7 to 28% [14, 15]. In addition, the identification of infected animals using routine diagnostic tests is imperfect. The sensitivity of available detection methods, such as faecal culture, polymerase chain reaction (PCR), or enzyme-linked immunosorbent assay (ELISA) is low [16], which leads to an underestimation of prevalence and to an “Iceberg effect” whereby the true prevalence in infected herds is greater than the apparent prevalence [17]. As a result, most infected animals, especially those in the early infection stage, remain undetected.

In this context, animal health managers need to find appropriate control measures to control Map spread in infected herds and prevent any further increase in prevalence. The main recommendations made by collective health managers are to improve calf rearing by limiting the contact between calves and infectious adults, and to eliminate or at least isolate animals detected as highly infectious [18–21]. Eliminating the offspring of highly infectious animals is also recommended. Nevertheless, assessing the effectiveness of complex strategies combining several control measures poses a real challenge, and animal health advisors and veterinarians must also be able to prioritize available measures in order to provide the most relevant, targeted recommendations to farmers based on their specific situation.

Modelling provides a suitable approach for addressing such an issue. Many models have been developed to represent Map spread within a dairy cattle herd [22–32], some to specifically assess the cost effectiveness of control measures such as test-and-cull [29, 33, 34], management and hygiene practices [19], and vaccination [25, 26]. Other modelling studies have focused on the cost-effectiveness of control programmes [27, 31] or their effect on within-herd disease transmission [32]. However, most existing models do not take Map survival within the environment explicitly into account, which is important because infection can occur if the environment is contaminated, even if no infected animal is present in the herd [35]. In addition, herd structure, which is known to greatly impact Map spread at the herd scale [23, 24], and the alternation of pasturing and housing are not always taken into consideration. Finally, only three existing models are individual-based [29, 30, 32], even though this would be required to design precise test-and-cull measures, with the possibility of culling each animal at a given date, according to its own characteristics or those of its dam. The three published individual-based models represent US dairy farms and the assumptions regarding herd characteristics differ considerably from those commonly observed in European dairy farming systems, such as a small to moderate herd size, structured by age, and using pasture for part of the year. Hence, a new individual-based model adapted to European farming systems is required for assessing control strategies in infected herds.

Our objective was to determine the most suitable measures among realistic ones that would be needed to prevent an increase of adult prevalence in infected dairy cattle herds by combining reduction of calf exposure to adult faeces and the test-and-cull of infectious animals. We formulated testing scenarios together with field partners (Animal Health Services from Brittany) to ensure that the tested scenarios are realistic and technically and economically feasible. We hypothesised that the initial prevalence might impair the effectiveness of a control measure, and that the relevance of different control measures might differ between herds with low versus high prevalence.

## Materials and methods

### General study design

A new individual-based model was designed to simulate Map spread within a dairy cattle herd and to assess the ability of control measures to maintain herd status within a range of prevalence. It combines population and infection dynamics and was suitably adapted to typical Western European dairy cattle herds. The focus was on control measures most commonly used in the field: calf rearing improvement (reduction of calf exposure to the environment contaminated by adult faeces) and test-and-cull of cows, with or without associated offspring removal and using a biased sex-ratio to maintain the female population despite a higher renewal. Our model includes an explicit survival of Map in the environment and can therefore accurately represent the reduction of calf exposure to adult faeces. Thanks to its individual-based nature, our model is able to represent precise test-and-cull strategy. Several initial herd statuses were defined according to the within-herd prevalence and various combinations of control measures were tested to identify the most influential for each status.

### Model description

A stochastic, time discrete, and individual-based model was developed in C++ language. Major assumptions were kept similar to those of a previously published compartmental model [23] which includes up to date knowledge on cattle paratuberculosis (infection, environment) and is adapted to Western European dairy cattle herds. Population and infection dynamics parameters were the same as previously described (Additional file 1). In our model, individual characteristics (described hereafter) were taken into account. Herd size was assumed to be maintained by internal renewal only and the purchase of cows was not modelled. Our focus was on already infected herds, therefore Map reintroduction into the herd was not considered. The model had a time step of 1 week, which is relevant for both Map infection dynamics and herd population dynamics, and was run over 15 years (780 weeks). A simulation was made of 1000 stochastic repetitions to ensure model output stability.

#### Population dynamics

**Figure 1:**
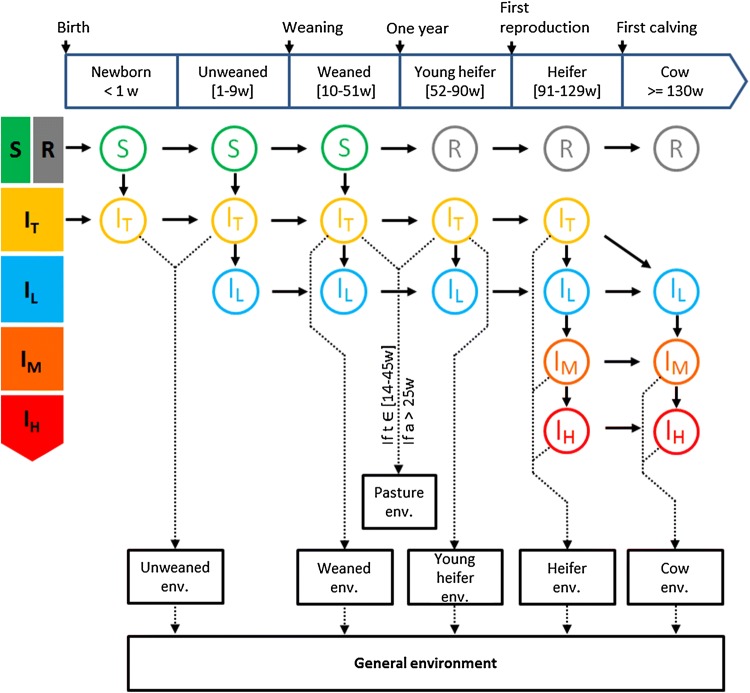
**Conceptual diagram of the model combining population and infection dynamics.** All health states are: susceptible (S), no longer susceptible (R), infected and transiently slightly infectious (I_T_), latently infected (I_L_), infected and moderately infectious (I_M_), and infected and highly infectious (I_H_). Solid arrows represent transitions between health states; dashed arrows represent the animals’ contribution to their local environment; w: week; t: week number in the year; a: age in weeks; env: environment.

Western European dairy cattle herds are generally structured according to age with young animals separated from adults [36]. Five age groups were considered (Figure 1) as typical of the structure of Western European dairy cattle herds: unweaned calves (up to 10 weeks of age), weaned calves (up to 1 year), young heifers (up to 91 weeks and not bred), bred heifers (up to first calving at 130 weeks of age), and cows (with known parity).

Population size was maintained by herd management. First, all male calves were assumed to be sold between the first and third week of age included, considering a sex-ratio of 50%. All female calves were kept. Births were managed with a year-round calving system. An age-based mortality rate was applied. Second, the culling rate of cows was updated according to the number of cows compared to a given threshold (*K*_*c*_, Additional file 2), accounting for parity (from one calving to more than five; Equation 1). The larger was the number of cows in the herd, the higher was the culling rate.

1 $$\sigma_{Pi}^{*} (t) = 1- exp\left( {\frac{{\sigma_{Pi} \cdot N_{c} (t)}}{{K_{c} }}} \right)$$

with σ_Pi_^*^(*t*) the updated culling rate of cows in parity *i* at time *t*, σ_*Pi*_ the reference culling rate of cows in parity *i*, *N*_*c*_(*t*) the total number of cows in the herd at time t, and *K*_*c*_ the threshold number of cows (Additional file 1). Third, heifers of 119 weeks of age (10 weeks before their first calving) could be sold at time *t* if the number of cows at time *t* exceeded the threshold.

The model also accounted for seasonality assuming a pasture period from April to mid-November, and a housing period for the remaining time. All animals older than 6 months were assumed to go on pasture, and young animals were raised on different pastures than adults.

#### Infection dynamics

Six mutually exclusive health states were modelled: susceptible (S), no longer susceptible (R), infected and transiently slightly infectious (I_T_, asymptomatic), latently infected without shedding (I_L_, asymptomatic), infected and moderately infectious (I_M_, asymptomatic), and infected and highly infectious (I_H_, could show clinical signs). Animal susceptibility was assumed to decrease exponentially with age. It was also assumed that, 1 year after birth, animals that had not been infected could no longer be infected, i.e. the possibility of infection as an adult was considered negligible as only already infected herds were studied.

Map survival in the environment was explicitly modelled. The amount of bacteria was updated at each time step, increasing with newly-shed bacteria and decreasing with Map death and hygiene measures (housing only). During the housing period (from mid-November to end of March), six environments explicitly represented the faecal contamination by Map of the animals’ living areas, one per age-based group (5 local environments; Figure 1), and one general farm environment (sum of the local environments). During the pasture period, unweaned calves and weaned calves less than 6 months old stayed inside and were still exposed to their local environment and to the general environment, while weaned calves more than 6 months old, young heifers, heifers, and cows went to their own respective pastures. Animals on pasture were not exposed to and did not contribute to the general environment during the pasture season, and were exposed only to their local pasture environment (assuming that weaned calves and young heifers were then raised together).

Five transmission routes were modeled: in utero transmission from I_L_, I_M_, and I_H_ dams with a higher rate of infection for calves born to I_H_ dams, indirect faecal-oral transmission due to the ingestion of contaminated faeces in the local (Equation 2) and general environments (Equation 3), and indirect transmission due to the ingestion of contaminated milk (only for unweaned calves and new-borns) and colostrum (only for new-borns). Infection transmission routes involving the ingestion of contaminated milk or colostrum was shown as minor transmission routes [23].

2 $$P_{l} = 1 - exp\left( {\frac{{ - exp\left( { - h \cdot age} \right) \cdot \beta_{l} \cdot E_{l} }}{{N_{l} \cdot \alpha }}} \right)$$

3 $$P_{g} = 1 - exp\left( {\frac{{ - exp\left( { - h \cdot age} \right) \cdot \beta_{g} \cdot e_{c} \cdot E_{g} }}{{N_{h} \cdot \alpha }}} \right)$$

with *P*_*l*_ and *P*_*g*_ the probabilities of infection from the local (age-based) and the general environments, respectively, *h* the coefficient of susceptibility which follows an exponential decrease with age, *α* the infectious dose, *β*_*l*_ the transmission rate if one infectious dose is present in the local environment (per week), *E*_*l*_ the amount of bacteria present in the age-specific local environment, *N*_*l*_ the number of animals present in that local environment, *β*_*g*_ the transmission rate if one infectious dose is present in the general environment (per week), *e*_*c*_ the factor of reduced calf exposure to the general environment (see “Control scenarios” section hereafter), *E*_*g*_ the amount of bacteria present in the general environment, and *N*_*h*_ the number of animals present in housing (i.e. not on pasture).

#### Individual characteristics and processes

Animals in the herd were considered as unique individuals with their own characteristics. They were defined according to their age (in weeks), health state, and parity (only for cows), as well as to possible test results (see “Control scenarios” section . Animals belonged to a given age group, which defined their contribution to local environmental contamination (shedding pattern linked to their health state), the probability that they would be infected by the various transmission routes, and the probability of calving (only for cows and bred heifers). All animals were linked to their dam, and all cows were linked to their calves: at each time *t*, the dam of each animal and the calves of each cow were known, so long as the animals were still present.

Each animal executed 2 phases each consisting of 3 processes. The first phase consisted of “shedding”, “calving”, and “aging” processes. The shedding process determined the amount of bacteria shed by each infected animal. The calving process managed new birth events. The aging process included growth and exit of animals (death, selling, culling). After this phase, all the environments were then updated. The second phase consisted of processes “transition”, “infection”, and “test” processes. The transition and infection processes were respectively related to the evolution of the health state after infection and to new animal infection. The test process corresponded to a detection test performed on targeted animals if required at time *t* (based on detection test frequency; see “Control scenarios” section hereafter).

### Initial conditions

Three initial herd statuses were defined based on the proportion of infected adults (i.e. adult prevalence), according to an unpublished study performed in Brittany (western France) by the regional Animal Health Services (GDS Bretagne): in low prevalence herds (status A) the apparent adult prevalence ranged from 0 to 3.5%, in moderate prevalence herds (status B) the apparent adult prevalence ranged from 3.5 to 10.5%, and in high prevalence herds (status C) the apparent adult prevalence ranged from 10.5 to 30%. In the model, the true prevalence was used. We based it on the Rogan–Gladen estimator [37] which estimates the true prevalence from the apparent prevalence, the test specificity and sensitivity (Equation 4).

4 $$True \;Prevalence = \frac{{Apparent\; Prevalence + \left( {Specificity - 1} \right)}}{{Specificity + \left( {Sensitivity - 1} \right)}}$$

Assuming a specificity of 1 and a sensitivity of about 0.5 in adults regardless of their health status, we transformed the observed categories based on apparent prevalence into true prevalence categories by multiplying the values by two. The true adult prevalence ranges for each herd status were the following: [0%; 7%[for herd status A, [7%; 21%[for herd status B, and [21%; 60%[for herd status C. Prevalence higher than 60% (herd status D) was not considered as such a high prevalence is rare and corresponds to herds in which no control measure has been implemented despite high losses and poor hygiene over a long time period.

**Figure 2:**
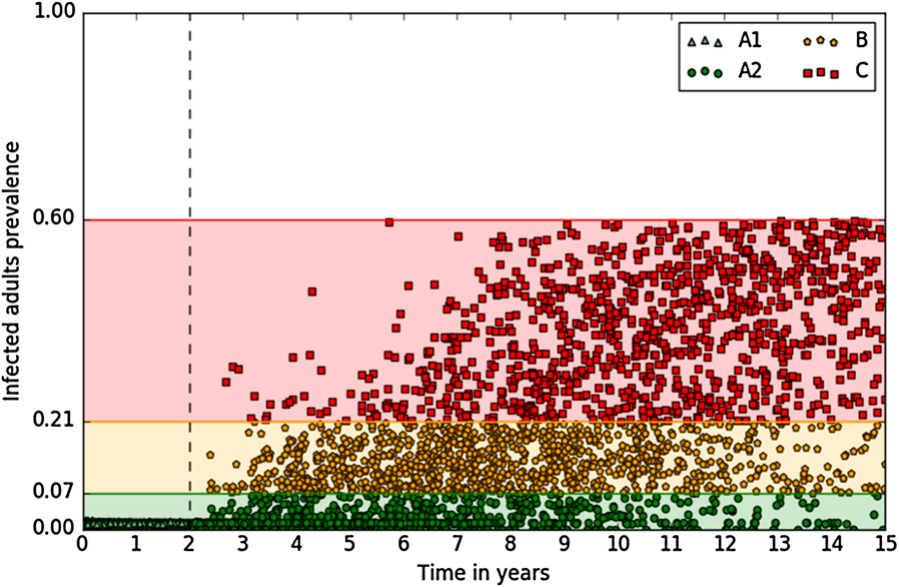
**Initial conditions of the individual-based model.** Four initial herd statuses were defined as early low (A1), low (A2), moderate (B), and high (C) prevalence of infected adults. There were 1000 initial conditions per herd status, the initial prevalence being distributed within the defined prevalence range and reached at variable times since Map introduction as predicted in the reference scenario (no control).

An innovative system was constructed to define the initial conditions corresponding to relevant situations in infected dairy cattle herds. For each herd status, 1000 possible starting points were defined that included all the required information about infected adult prevalence, headcount per age group and health state, and the associated amount of bacteria in each environment (housing and pasture) (Figure 2). Selection of the starting points was based on repetitions of our reference scenario, which corresponded to the introduction of a single infected heifer near to calving (I_M_) into a naïve herd (no reintroduction, closed herd) without any control measure being implemented. First, 5000 repetitions of this reference scenario were conducted over 15 years to get a large amount of possible prevalence trajectories. After MAP was first introduced, there was no clear epidemic phase followed by a steady prevalence. Instead, herds could experience highly variable prevalence trajectories despite similar transmission parameters and management practices (few trajectories are shown in Additional file 2). Hence, we assumed that infected herds in a given herd status could have any of the possible distributions of animals per age group and health state as predicted regardless of time since infection. A total of 3 905 000 points was obtained, corresponding to weekly pictures of infected herds. As points before 2 years represented situations mostly influenced by the initial introduction of Map, herd status A was split into two: early low prevalence (status A1) for points before 2 years, and low prevalence (status A2) for points after 2 years. One thousand points for each initial herd status (A1, A2, B, and C) were selected at random within the relevant ranges in this distribution (Figure 2).

### Control scenarios

First, calf rearing improvement was defined as a reduced exposure to the general environment, mostly contaminated by adults, by varying parameter *e*_*c*_ of function *P*_*g*_, and considering four values ranging from 1.0 (default value, no improvement) to 0.35 (almost divided by 3; Table 1). Such a decreased probability of infection due to contacts with the general environment would correspond either to improved hygiene or to a reduction in calf-to-adult contacts.

**Table 1:** **Model parameters related to control measures**

Parameters	Values
Reduction of calf exposure to the general environment	[1.0, 0.65, 0.5, 0.35]
Test frequency	[None, 104, 52] in weeks
Test sensitivity per health state [38]	
I_M_ animals	0.47
I_H_ animals	0.71
Culling delay of animals detected as	
Moderately positive (I_M_)	26 weeks
Highly positive (I_H_)	[4, 13] in weeks
Culling proportion of animals detected as	
Moderately positive (I_M_)	[0%, 50%]
Highly positive (I_H_)	100%
Sex-ratio (female side)	[0.5, 0.6, 0.7]
Selling proportion of marked calves	[0.0, 0.5, 0.65, 0.8]

Second, we formulated several realistic and technically and economically feasible testing scenarios with Animal Health Services from Brittany. Test-and-cull strategies were implemented using the individual-based feature of the model. As all animals were individually represented with their own characteristics, it was possible to perform a detection test on specific animals and to cull each of them according to their test result. Cows have the highest probability to be in one of the late stages of infection (I_M_ and I_H_) which are the most important shedding stages and more easily detectable. Hence, detection tests were performed only on cows (at 1^st^ January) and corresponded to a serum antibody ELISA test. Three possible test frequencies were considered (never, every 2 years, and each year). Test sensitivity per health state was derived from [38]: 0.15 for I_T_ and I_L_, 0.47 for I_M_, and 0.71 for I_H_. Test specificity was assumed to be 0.985. Four possible test results were considered: negative, slightly positive, moderately positive, and highly positive. The test-and-cull strategy implemented in the model targeted only moderately and highly positive animals. In the absence of precise knowledge, I_M_ cows detected as positive were assumed to be moderately positive, while I_H_ cows detected as positive were assumed to be highly positive. All *R*, I_T_, and I_L_ cows detected as positive were assumed to be only slightly positive. Delay and proportion of culling were defined for each test result (Table 1). Calves born to dams detected as positive are more likely to be infected in utero, so their removal with their dam is sometimes recommended. We used the test results of dams to remove such calves: calves born to dams detected as highly positive during their first 10 weeks of life were marked and sold at 21 weeks of age (~150 days) according to the selling proportion of marked calves (Table 1). The model also included a sexing option to modify the sex-ratio at birth and have more female calves and thus avoid a decreased headcount due to animal culling (Table 1).

All the parameter combinations were covered, resulting in a total of 388 scenarios including the reference scenario with no control measures (1 scenario), scenarios with only calf rearing improvement (3 scenarios), only test-and-cull (96 scenarios), and both measures simultaneously (288 scenarios). All initial herd statuses (A_1_, A_2_, B, and C), were taken into account and 1552 scenarios were explored.

### Outputs and statistical treatment

Two model outputs were considered: the persistence of infection in the herd, a binary output with a value of 1 if at least one infected animal was present in the herd and/or if bacteria were present in the environment, otherwise a value of 0; the true adult prevalence (ranging from 0 to 1). Both outputs were considered at three time points: years 5, 10, and 15. The concept of “non-degrading herd status” was defined as a herd being in the same or in a better (i.e. lower prevalence) herd status after 5, 10, and 15 years. The probability that the initial herd status would not degrade over time was computed by considering the 1000 repetitions per scenario. This probability corresponded to the percentage of repetitions that presented a true adult prevalence related to the same or to a better herd status. Here, we considered the extinction of infection (persistence equal to 0) to be better than herd status A as no reintroduction was assumed, and herd status D (adult prevalence above 60%) to be worse than herd status C.

The most important parameters involved in control measures which would explain the probability of non-degrading herd status were identified by performing statistical discriminant analyses using the *Python* library called *scikit*-*learn* and *Random Forest Classifier* (RF), a machine learning based method. This method makes it possible to build predictive statistical models using explanatory variables (parameters of control measures in our case) with the aim of predicting a given variable (probability of non-degrading herd status). Predictive statistical models are defined by their precision and the relative importance values (as a percentage) obtained for each given variable during learning.

Since the reduction of calf exposure to the adult environment parameter was linked to the main routes of infection [31], each of its values was considered independently and excluded from discriminant analyses to avoid neglecting the others (related to test-and-cull). In this way, the combined effects of reducing calf exposure and test-and-cull on the probability of non-degrading herd status could be determined by performing statistical analyses on 4 (all possible values for calf exposure) samples of 96 scenarios (number of scenarios when only test-and-cull parameters were varied). The probability of non-degrading herd status was categorized with a score ranging from 0 to 2, these scores corresponding respectively to a probability between percentile 0 and 33, 33 and 66%, 66 and 100%. In total, 48 predictive statistical models were analysed, based on each initial herd status, each considered year, and all values of calf exposure.

The most suitable parameters of the RF method were selected for each by cross-validation. These parameters were the number of trees in the forest (from 50 to 100) and the number of random features to consider (from 2 to 5). The cross-validation involved 10 stratified samples of scenarios and randomly split into 70% for model training and 30% for precision model testing. The most suitable RF parameters were selected according to the average precision obtained by cross-validation and saved.

All the predictive statistical models were then trained and tested as in the previous step on stratified and random samples of scenarios (70 and 30% respectively). Each model was assigned a training accuracy to check the quality of the learning and a test accuracy to control its extrapolation to another dataset. Predictive models with an accuracy above 70% are considered to be accurate enough to explain the probability of non-degrading herd status. Predictive models with an accuracy of below 50% are considered worse than a totally random model.

We then restricted our analyses to those parameters which best explained the probability of non-degrading herd status. Test-and-cull parameters with a greater relative importance than the threshold of 20% were considered as the most influential. The reduction of calf exposure was also included to the selected parameters. Only those scenarios which varied these selected parameters and used standard values for all other parameters were selected. For each combination of initial herd status (A1, A2, B, and C) and year (5, 10, and 15), a rank was computed and assigned to each scenario according to its probability of non-degraded herd status. For a given status-year combination, rank one was given to the scenario with the highest probability of non-degrading herd status. All scenarios were assigned a total of 12 ranks (one per combination of initial herd status and year). We also calculated an overall rank per scenario from the sum of the ranks over herd status-year combinations for which the predictive models were sufficiently accurate (>70%). The overall rank was normalized to range from 1 (best) to a maximum defined by the number of restricted scenarios.

## Results

### Probability of non-degrading herd status

**Figure 3:**
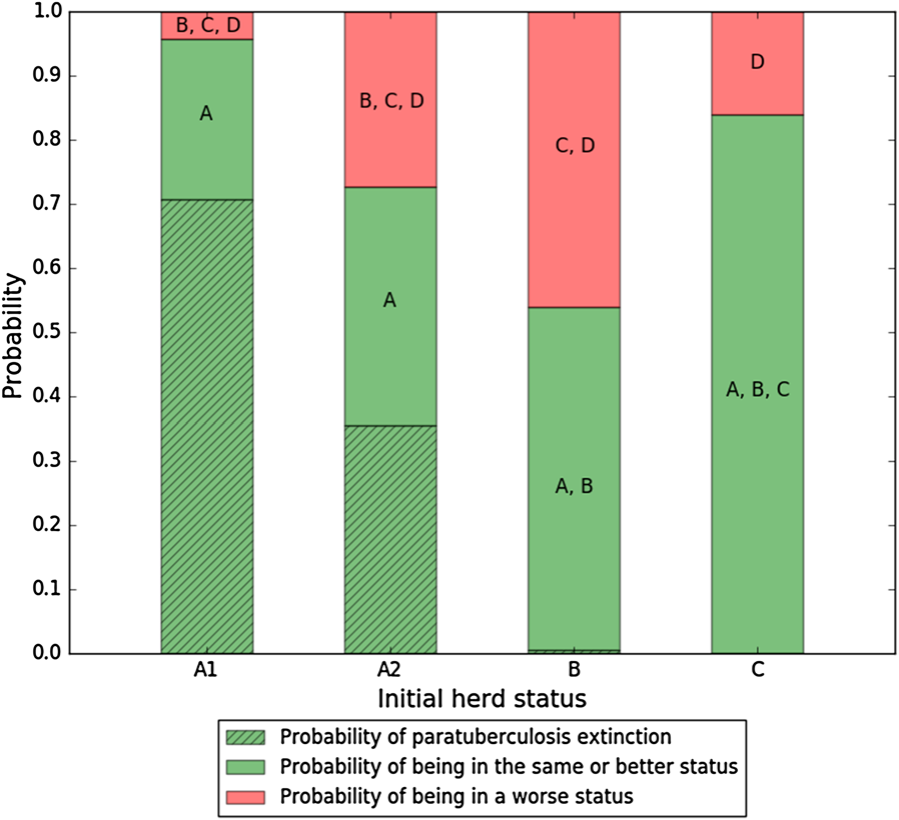
**Probability of non-degrading herd status over a 5-year period with maximum efforts regarding control measures.** Initial herd statuses are defined as early low (A1), low (A2), moderate (B), and high (C) adult prevalence. Herd status D corresponds to adult prevalence above 60%. Paratuberculosis extinction implies no more infected animals and no more bacteria in the living environments.

The scenario involving the maximum control effort in this study was defined by a calf exposure to cow environment almost divided by 3, a test performed on cows every year, culling half of the moderately positive animals at most 6 months after infection, culling all highly positive animals at most 1 month after infection, and a removal rate of 80% for marked calves born to dams detected as highly positive during the calves’ first 10 weeks of life. For this scenario, the model predicted a probability of non-degrading herd status above 0.70 for low and high prevalence herd statuses (A and C) compared with about 0.5 for moderate ones (B) (Figure 3). For the low prevalence herd status, Map extinction was highly probable.

### Measures needed to prevent degrading herd status

All the predictive statistical models using the RF method presented a training accuracy near or equal to 100% and a testing accuracy above 70%, except for combination A1 at year 5 (Additional file 3). For this combination, and regardless of calf exposure, we were unable to build an accurate predictive model as the probability of non-degrading herd status always remained high and barely variable (0.84–0.96). No further analysis was therefore possible for combination A1 at year 5. For all the other combinations, predictive models were able to explain the probability of non-degrading herd status with the control measures implemented in the scenarios.

**Figure 4:**
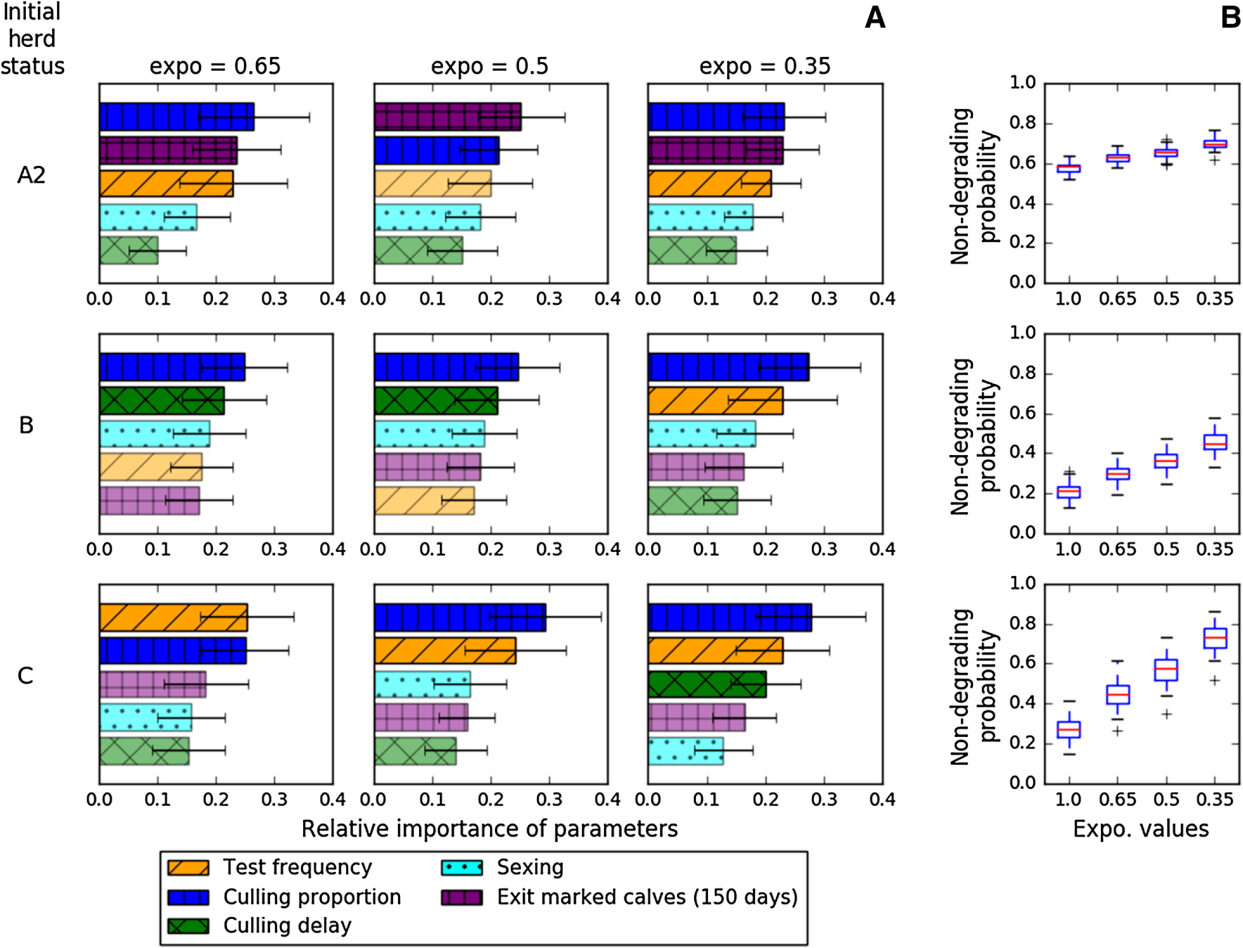
**Influence of control measures modalities on the probability of non-degrading herd status over a 5-year period. A** Relative importance of each test-and-cull parameter linked to the predictive statistical models built with *Random Forest Classifier* method, and **B** the associated probability of non-degrading herd status over a 5-year period and according to the initial herd status (A2, B, and C, in lines) and the reduction of calf exposure (expo = 0.65, 0.5, and 0.35, in columns).

**Figure 5:**
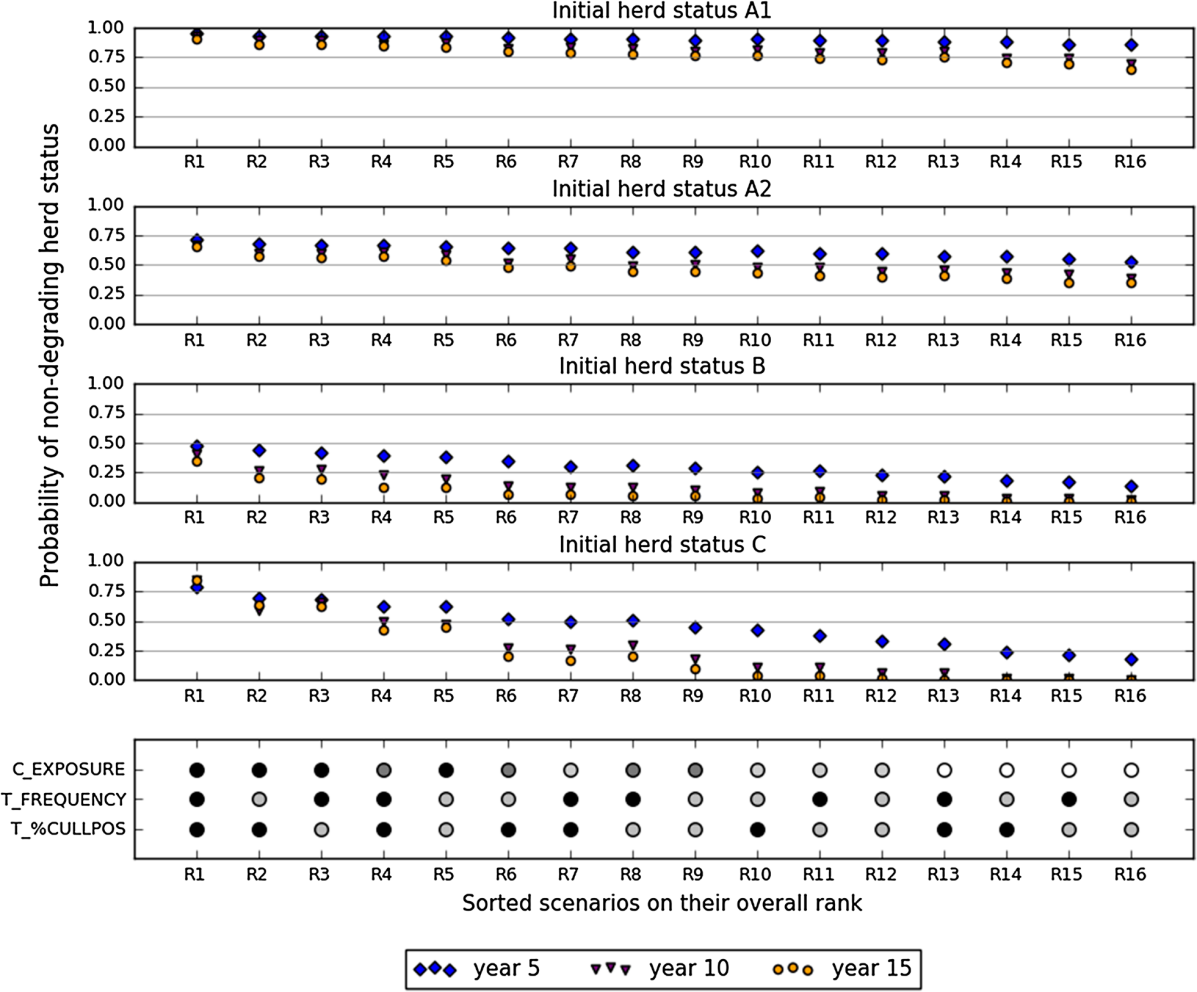
**Probability of non-degrading herd status over a 5-, 10-, and 15-year period according to the initial herd status (in lines).** The figure shows only selected scenarios (16), sorted by their overall rank. Parameter values are shown as circles. Parameter of reducing calf exposure (C_EXPOSURE) has four possible values ranging from 0.35 (optimal, plain circle) to 1.0 (standard, empty circle). Other parameters (test frequency as T_FREQUENCY, and culling proportion as T_%CULLPOS) have only two possible values (see Table 1). Scenarios with the best overall ranks are on the left, scenarios with the worst ones on the right.

In the short term (year 5), those test-and-cull parameters that had been highlighted as the most influential differed according to the initial herd status and to the reduction of calf exposure to the adults’ environment (Figure 4). For herds with a low prevalence status (A2), the two most influential parameters, irrespective of calf exposure, were the culling proportion of moderately positive animals and the removal of marked calves, followed by test frequency. For herds with a moderate prevalence status (B), herds achieving a small to moderate decrease in calf exposure should increase the culling proportion of moderately positive animals and decrease the culling delay of highly positive animals, while herds with a highly reduced calf exposure should focus on culling proportion and test frequency. Finally, the two most influential parameters for herds with a high prevalence status (C) were test frequency and culling proportion of highly positive animals as in A2, while the removal of marked calves or the culling delay were no longer relevant. Sexing was never influential. Figure 5 also confirmed the impact of reducing calf exposure to the adults’ environment on the probability of non-degrading herd status, especially for initial herd statuses B and C.

In the longer term (years 10 and 15), the two most influential test-and-cull parameters were always the culling proportion of moderately positive animals and the test frequency (Additional files 4 and 5). The culling delay of highly positive animals—for the range of values tested in this study, i.e. with a maximum of 3-month delay—and the removal of marked calves were not influential in the long term, irrespective of the initial herd status. As expected, the results for initial herd status A1 at year 10 were similar to those obtained for initial status A2 at year 5.

In the following sections, only the two most influential test-and-cull parameters that showed up in almost all the combinations are considered, namely test frequency and culling proportion of moderately positive animals, coupled with calf exposure.

### Scenarios ensuring a high probability of non-degrading herd status

Sixteen scenarios were selected to explore the three most influential parameters (reducing calf exposure, test frequency, and test culling proportion) in greater depth, while maintaining the other parameters at their default values (Figure 5). The eleven herd-status-year combinations for which the statistical models were sufficiently accurate (all except for A1 at year 5) were used to calculate the overall rank of each of the 16 scenarios. The selected scenarios were ordered by increasing overall rank. The scenario with an overall rank of 1 represented the first rank in all herd-status-year combinations. As expected this scenario corresponded to the greatest reduction of calf exposure, the highest test frequency (every year), and the highest culling proportion (culling 50% of moderately positives). It corresponded to the maximum possible effort when implementing control measures with the selected parameters. The scenario with overall rank of 16 corresponded to no reduction of calf exposure and the minimum possible effort regarding test-and-cull strategy.

All scenarios with the lowest calf exposure were in the five best scenarios. Furthermore, all scenarios with the default value of this parameter were in the four worst scenarios, thus confirming the crucial role of this measure in achieving a non-degrading herd status. The scenario with an overall rank 4 presented the best values for test-and-cull parameters (Table 1) but only a halved calf exposure. Considering the same test-and-cull parameter values, scenarios with calf exposure values of 0.65 and 1.0 presented overall ranks of 7 and 13 respectively. The test-and-cull strategy alone was not sufficient to attain the highest overall ranks, even with the best parameter values. On the contrary, the scenario with the lowest calf exposure and the worst test-and-cull parameter values only attained an overall rank of 5. Comparison of this scenario to the one with overall rank of 1 illustrated the added-value of combining both measures to improve the probability of non-degrading herd status.

### Does the probability of non-degrading herd status persist over time?

The probability of non-degrading herd status decreased for all scenarios (except one, C with the scenario with an overall rank of 1) over time, especially between year 5 and year 10 irrespective of the initial herd status. The decrease between year 10 and year 15 was always less visible. For initial herd status A1, the probabilities were above 0.75, regardless of time, for the first ten scenarios. For initial herd status B, the probability of non-degrading herd status decreased gradually and directly from the scenario with an overall rank of 2 to the last one.

## Discussion

### Implication of main findings

Our new individual-based model renders it possible to account for the heterogeneity of within-herd prevalence among infected herds when implementing control measures in dairy cattle herds. We show that the probability to prevent the increase in paratuberculosis prevalence in dairy cattle herds largely varies with adult prevalence, and that most relevant test-and-cull options to combine with calf management depends on herd prevalence status.

First, we confirmed that the probability of non-degrading herd status over time differed according to the initial herd prevalence status. The results obtained for early infected herds clearly differed from the others. In most cases, newly infected herds cannot be detected by routine tests because mostly young animals are infected. Our results show that when naive herds were infected after the purchase of an infected animal, the probability of the infection to fade out spontaneously was high, in agreement with previous results [23]. Nevertheless, if Map can be reintroduced into herds, e.g. through animal purchases [39, 40], such herds would be expected to rapidly have a higher prevalence. Control measures should therefore be implemented in herds with a high probability of being uninfected but located in an infected region. After 2 years, if the herd was still infected, it appeared to be very difficult to stabilize its status. It was even worst for moderate prevalence herds, even if the best control options were implemented. The situation in high prevalence herds might be stabilized by implementing maximum (but realistic) efforts on control measures. However, the probability of the prevalence worsening over time remained non-negligible.

**Figure 6:**
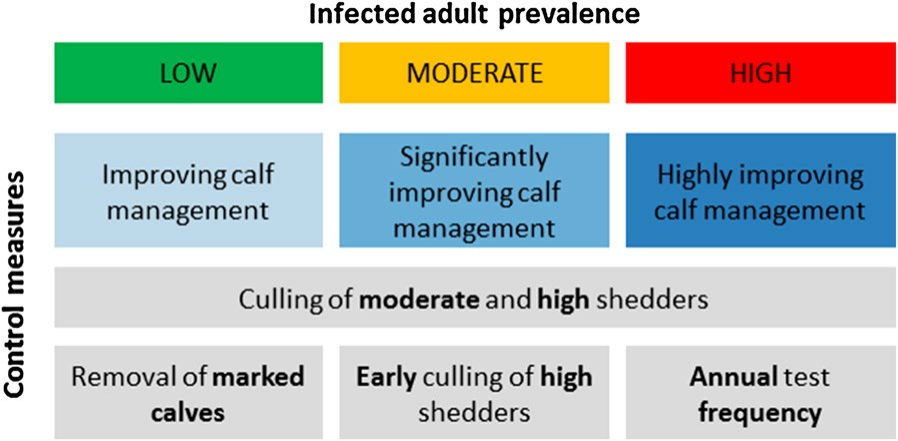
**Summary of prioritized control measures on the short term (5** **years) according to the prevalence in infected adults.** Control strategies combine an improved calf management through reducing calf exposure to adult environments (always recommended but at various levels of implementation) and test-and-cull options (whose relevance varies with adult prevalence).

Second, we confirmed the effectiveness of combining calf rearing improvement with test-and-cull of adults [19], based on epidemiological criteria, and demonstrate that test-and-cull options should be adapted to herd prevalence status (Figure 6). Culling moderate and high shedders was always retained, but further control options changed with prevalence. There was a balance between the removal of future shedders (marked calves), of high shedders, and of moderate shedders (with more frequent tests to detect them). In the Netherlands, calves born to dams with clinical symptoms should be culled [41]. According to our results, calf removal is only of interest in low prevalence herds. In such herds, very few adults will be detected as infected and removing their calves is relevant to limit the occurrence of future shedders. In addition, this option will be economically viable for health advisors as their number will remain low. Decreasing the delay in culling animals detected as high shedders was only effective in moderate prevalence herds, where many of the infected animals cannot be detected (too young, low shedding levels, etc.). In these herds, high shedders are not numerous, thus priority given to their culling appears to be practically feasible. Culling delays longer than 3 months have not been tested as it would increase the time window for high shedders to contribute to environmental contamination. Finally, focusing on test frequency appeared to be a better option than reducing culling delay in high prevalence herds. On a longer term, the most effective test-and-cull options to combine with a reduction of calf exposure to the adults’ environment is to cull a higher proportion of detected infected animals and to increase test frequency. It did not vary with the initial herd status and corresponded to the same option as for high prevalence herds in the short term.

The role of reducing calf exposure was already largely demonstrated [18, 42] and also shown in another modelling study [20]. If this control measure was not applied, the probability of stabilizing herd status over time was very low. Similarly, it was known that implementing a test-and-cull strategy alone, even with considerable effort, was not sufficient to prevent a degradation of herd status, as shown in previous modelling studies [33, 40, 43]. Nevertheless, it is not always possible to modify calf exposure on farms because it can involve expensive changes in farm structure (an additional building), that most farmers cannot afford. We show that in such a case, decreasing the delay before culling high shedders would be a good option, but only for moderate prevalence herds.

### Model assumptions

The individual-based feature of the model enabled us to apply realistic calf rearing improvements and feasible test-and-cull strategies, as close as possible to the ones recommended in France by animal health advisors. Not all animals detected as positive experienced the same consequences, which were defined for each animal based on their true health state, the culling proportion of moderately positive animals, and the culling delay of highly positive animals. In this way, the animal exits from the herd could be realistically modelled and were more in line with the farmer’s prioritisation of animals to be culled.

Our individual-based model was calibrated on typical Western Europe dairy cattle herds, with an age structure, alternating periods at pasture and in housing, a high cow renewal rate, and few cow purchases. For such a farming system, the stochastic feature of the model already leads to heterogeneous prevalence trajectories since first introduction of MAP, which was accounted for through an innovative system for defining initial conditions. Conclusions might differ for other farming systems, especially those with more contacts between adults and young animals, or with a lower transmission rate due to extensive farming conditions. Additional data then are required for calibrating the model accordingly, as we made previously using French data [23]. In addition, no data is currently available about costs associated with most measures (calf management, hygiene, etc.), as well as with disease impact on production and farmer’s work. Therefore, we used only epidemiological criteria to compare scenarios and we collaborated with field partners (Animal Health Services from Brittany) to formulate control scenarios that can be considered as realistic and technically and economically feasible based on their field expertise. The sexing option in the test-and-cull strategy was not influential based on epidemiological criteria. As its objective is to prevent a decreased headcount due to animal culling, it could be influential however if economic criteria were to be considered.

Other major model assumptions concern infection dynamics: no infection of adults and high heterogeneity in shedding levels between and within health states. Adult infection has been demonstrated in animals that were born in naïve herds and subsequently infected once exposed as an adult to a large infectious dose [3]. Alternative epidemiological models include this assumption [29, 32]. However, animals born in an infected herd and still not infected as adults have a very low probability of being infected, justifying our decision to neglect adult infection here. The shedding heterogeneity was calibrated based on literature [23] and explained a large part of the stochastic prevalence trajectories. Cows can shed a large amount of bacteria on a single occasion, followed by a lower shedding rate during another time period. Such a shedding heterogeneity was accounted for in the model. Nevertheless, the effect of such a shedding heterogeneity on test results was not fully accounted for in the absence of a proven association between the shedding level and the level of positivity to detection tests. If such data was available, the individual-based feature of the model would enable us to refine test-and-cull options.

In conclusion, all infected herds need to be detected as soon as possible in order to implement calf rearing improvement and test-and-cull. These control options should be adapted according to herd prevalence status. Moderate prevalence herds are the critical ones, noting that the most rigorous combination of control measures only ensured a probability of non-degrading herd status of about 50%. In contrast, for high prevalence herds, a high probability of non-degrading herd status (from 80 to 95%) could be achieved. A further increase in prevalence in already high prevalence herds can be prevented by implementing realistic control measures. Indeed, in these herds, prevalence should not exceed 60% if rigorous control measures are implemented, as currently undertaken in heavily infected herds in the field.

## Additional files

**Additional file 1.** **Model parameters for processes related to population and infection dynamics.** Value and source for each parameter of the population and infection dynamics processes.

**Additional file 2.** **Sample of 11 trajectories of infected adult prevalence obtained with the reference scenario.** Reference scenario was defined as the introduction of an infected heifer (*I*_*M*_) in a naïve herd and no control implementation. Each trajectory represents the variation of infected adult prevalence over time since Map introduction. The sample of 11 trajectories has been extracted from the 5000 trajectories used to build initial conditions. Two trajectories (blue and brown) have been highlighted to illustrate model stochasticity and the absence of an early epidemic phase followed by a steady-state prevalence on the contrary to what is classically encountered in epidemiology.

**Additional file 3.** **Precision of predictive statistical models built with the** ***Random Forest Classifier*** **method**. All combinations of initial herd status (early low adult prevalence: A1; low adult prevalence: A2; moderate adult prevalence: B; high adult prevalence: C), year (5, 10, and 15 years), and calf exposure (no reduction: expo = 1; and 3 levels of reduction: expo = 0.65, 0.5, and 0.35) are shown. Accuracies are considered as good enough when above 70%.

**Additional file 4.** **Influence of control measure modalities on the probability of non-degrading herd status over a 10-year period**. (Panel A) Relative importance of each test-and-cull parameter linked to the predictive statistical model built with the *Random Forest Classifier* method, and (panel B) associated probability of non-degrading herd status over a 10-year period and according to initial herd status (A2, B, and C, in lines) and reduction of calf exposure (expo = 0.65, 0.5, and 0.35, in columns).

**Additional file 5.** **Influence of control measure modalities on the probability of non-degrading herd status over a 15-year period**. (Panel A) Relative importance of each test-and-cull parameter linked to the predictive statistical model built with the *Random Forest Classifier* method, and (panel B) associated probability of non-degrading herd status over a 15-year period and according to initial herd status (A2, B, and C, in lines) and reduction of calf exposure (expo = 0.65, 0.5, and 0.35, in columns).

## Abbreviations

MAP: *Mycobacterium avium* subsp. *Paratuberculosis*
ELISA: enzyme-linked immunosorbent assay
RF: *Random Forest Classifier*

## References

[1] Sweeney RW, Collins MT, Koets AP, Mcguirk SM, Roussel AJ (2012). Paratuberculosis (Johne’s disease) in cattle and other susceptible species. J Vet Intern Med.

[2] Elliott GN, Hough RL, Avery LM, Maltin CA, Campbell CD (2015). Environmental risk factors in the incidence of Johne’s disease. Crit Rev Microbiol.

[3] Windsor PA, Whittington RJ (2010). Evidence for age susceptibility of cattle to Johne’s disease. Vet J.

[4] Mortier RAR, Barkema HW, Bystrom JM, Illanes O, Orsel K, Wolf R, Atkins G, De Buck J (2013). Evaluation of age-dependent susceptibility in calves infected with two doses of *Mycobacterium avium* subspecies *paratuberculosis* using pathology and tissue culture. Vet Res.

[5] Whittington RJ, Windsor PA (2009). In utero infection of cattle with *Mycobacterium avium* subsp. *paratuberculosis*: a critical review and meta-analysis. Vet J.

[6] van Roermund HJW, Bakker D, Willemsen PTJ, de Jong MCM (2007). Horizontal transmission of *Mycobacterium avium* subsp. *paratuberculosis* in cattle in an experimental setting: calves can transmit the infection to other calves. Vet Microbiol.

[7] Mortier RAR, Barkema HW, De Buck J (2015). Susceptibility to and diagnosis of *Mycobacterium avium* subspecies *paratuberculosis* infection in dairy calves: a review. Prev Vet Med.

[8] Mortier RAR, Barkema HW, Orsel K, Wolf R, De Buck J (2014). Shedding patterns of dairy calves experimentally infected with *Mycobacterium avium* subspecies *paratuberculosis*. Vet Res.

[9] Weber MF, Kogut J, de Bree J, van Schaik G, Nielen M (2010). Age at which dairy cattle become *Mycobacterium avium* subsp. *paratuberculosis* faecal culture positive. Prev Vet Med.

[10] Mitchell RM, Schukken Y, Koets A, Weber M, Bakker D, Stabel J, Whitlock RH, Louzoun Y (2015). Differences in intermittent and continuous fecal shedding patterns between natural and experimental *Mycobacterium avium* subspecies *paratuberculosis* infections in cattle. Vet Res.

[11] Tiwari A, VanLeeuwen JA, McKenna SLB, Keefe GP, Barkema HW (2006). Johne’s disease in Canada Part I: clinical symptoms, pathophysiology, diagnosis, and prevalence in dairy herds. Can Vet J.

[12] Ott SL, Wells SJ, Wagner BA (1999). Herd-level economic losses associated with Johne’s disease on US dairy operations. Prev Vet Med.

[13] Behr MA, Collins DM (2010). Paratuberculosis: organism, disease, Control.

[14] Good M, Clegg T, Sheridan H, Yearsely D, O’Brien T, Egan J, Mullowney P (2009). Prevalence and distribution of paratuberculosis (Johne’s disease) in cattle herds in Ireland. Ir Vet J.

[15] Raizman EA, Wells SJ, Muñoz-Zanzi CA, Tavornpanich S (2011). Estimated within-herd prevalence (WHP) of *Mycobacterium avium* subsp. *paratuberculosis* in a sample of Minnesota dairy herds using bacterial culture of pooled fecal samples. Can J Vet Res.

[16] Nielsen SS, Toft N (2008). Ante mortem diagnosis of paratuberculosis: a review of accuracies of ELISA, interferon-g assay and faecal culture techniques. Vet Microbiol.

[17] Magombedze G, Ngonghala CN, Lanzas C (2013). Evalution of the “Iceberg Phenomenon” in Johne’s disease through mathematical modelling. PLoS One.

[18] McKenna SLB, Keefe GP, Tiwari A, VanLeeuwen J, Barkema HW (2006). Johne’s disease in Canada part II: disease impacts, risk factors, and control programs for dairy producers. Can Vet J.

[19] Dorshorst NC, Collins MT, Lombard JE (2006). Decision analysis model for paratuberculosis control in commercial dairy herds. Prev Vet Med.

[20] Kudahl A, Nielsen S, Østergaard S (2008). Economy, efficacy, and feasibility of a risk-based control program against paratuberculosis. J Dairy Sci.

[21] Khol JL, Damoser J, Dünser M, Baumgartner W (2007). Paratuberculosis, a notifiable disease in Austria—current status, compulsory measures and first experiences. Prev Vet Med.

[22] Marcé C, Ezanno P, Weber MF, Seegers H, Pfeiffer DU, Fourichon C (2010). Invited review: modeling within-herd transmission of *Mycobacterium avium* subspecies *paratuberculosis* in dairy cattle: a review. J Dairy Sci.

[23] Marcé C, Ezanno P, Seegers H, Pfeiffer D, Fourichon C (2011). Predicting fadeout versus persistence of paratuberculosis in a dairy cattle herd for management and control purposes: a modelling study. Vet Res.

[24] Marcé C, Ezanno P, Seegers H, Pfeiffer DU, Fourichon C (2011). Within-herd contact structure and transmission of *Mycobacterium avium* subspecies *paratuberculosis* in a persistently infected dairy cattle herd. Prev Vet Med.

[25] Lu Z, Schukken YH, Smith RL, Mitchell RM, Gröhn YT (2013). Impact of imperfect *Mycobacterium avium* subsp. *paratuberculosis* vaccines in dairy herds: a mathematical modeling approach. Prev Vet Med.

[26] Lu Z, Schukken YH, Smith RL, Gröhn YT (2013). Using vaccination to prevent the invasion of *Mycobacterium avium* subsp. *paratuberculosis* in dairy herds: a stochastic simulation study. Prev Vet Med.

[27] Cho J, Tauer LW, Schukken YH, Smith RL, Lu Z, Gröhn YT (2013). Cost-effective control strategies for Johne’s disease in dairy herds. Can J Agric Econ.

[28] Smith RL, Schukken YH, Gröhn YT (2015). A new compartmental model of *Mycobacterium avium* subsp. *paratuberculosis* infection dynamics in cattle. Prev Vet Med.

[29] Robins J, Bogen S, Francis A, Westhoek A, Kanarek A, Lenhart S, Eda S (2015). Agent-based model for Johne’s disease dynamics in a dairy herd. Vet Res.

[30] Al-Mamun MA, Smith RL, Schukken YH, Gröhn YT (2016). Modeling of *Mycobacterium avium* subsp. *paratuberculosis* dynamics in a dairy herd: an individual based approach. J Theor Biol.

[31] Smith RL, Al-Mamun MA, Gröhn YT (2017). Economic consequences of paratuberculosis control in dairy cattle: a stochastic modeling study. Prev Vet Med.

[32] Al-Mamun MA, Smith RL, Schukken YH, Gröhn YT (2017). Use of an individual-based model to control transmission pathways of *Mycobacterium avium* subsp. *paratuberculosis* infection in cattle herds. Sci Rep.

[33] Lu Z, Mitchell RM, Smith RL, Van Kessel JS, Chapagain PP, Schukken YH, Grohn YT (2008). The importance of culling in Johne’s disease control. J Theor Biol.

[34] Lu Z, Schukken YH, Smith RL, Gröhn YT (2010). Stochastic simulations of a multi-group compartmental model for Johne’s disease on US dairy herds with test-based culling intervention. J Theor Biol.

[35] Whittington RJ, Marshall DJ, Nicholls PJ, Marsh IB, Reddacliff LA (2004). Survival and dormancy of Map in the environment. Appl Environ Microbiol.

[36] Marcé C, Guatteo R, Bareille N, Fourichon C (2010). Dairy calf housing systems across Europe and risk for calf infectious diseases. Animal.

[37] Rogan WJ, Gladen B (1978). Estimating prevalence from the results of a screening test. Am J Epidemiol.

[38] More SJ, Cameron AR, Strain S, Cashman W, Ezanno P, Kenny K, Fourichon C, Graham D (2015). Evaluation of testing strategies to identify infected animals at a single round of testing within dairy herds known to be infected with *Mycobacterium avium* ssp. *paratuberculosis*. J Dairy Sci.

[39] Beaunée G, Vergu E, Ezanno P (2015). Modelling of paratuberculosis spread between dairy cattle farms at a regional scale. Vet Res.

[40] Beaunée G, Vergu E, Joly A, Ezanno P (2017). Controlling bovine paratuberculosis at a regional scale: towards a decision modelling tool. J Theor Biol.

[41] Benedictus G, Verhoeff J, Schukken Y, Hesselink J (1999) Dutch Paratuberculosis Program: history, principles and development. In: Proceedings of 6^th^ International Colloquium of Paratuberculosis, vol 77, p. 9–21 10.1016/s0378-1135(00)00325-4 11118725

[42] Ridge SE, Heuer C, Cogger N, Heck A, Moor S, Baker IM, Vaughan S (2010). Herd management practices and the transmission of Johne’s disease within infected dairy herds in Victoria, Australia. Prev Vet Med.

[43] Groenendaal H, Galligan DT (2003). Economic consequences of control programs for paratuberculosis in midsize dairy farms in the United States. J Am Vet Med Assoc.

