# Supplementary material for: Control measures to prevent the increase of paratuberculosis prevalence in dairy cattle herds: an individual-based modelling approach
Source: Vet Res. 2018 Jul 13;49:60. doi: 10.1186/s13567-018-0557-3 (PMC6044053; doi:10.1186/s13567-018-0557-3)
Supplement: Supplementary file 1 — Additional file 1. Model parameters for processes related to population and infection dynamics. Value and source for each parameter of the population and infection dynamics processes. [file 13567_2018_557_MOESM1_ESM.docx]

**Additional file 1 Model parameters for processes related to population dynamics.**

| **Parameters** | **Values** | **Definition** | **Source** |
| --- | --- | --- | --- |
| *σ_B_* | 0.07 | Calf mortality rate at birth | ^a^, [23] |
| *σ_m_* | 0.206 | Exit rate of male calves, weeks 2 to 4 (per week) | [23] |
| *σ_C1_* | 0.015 | Death rate of female calves, weeks 1 and 2 (per week) | [23] |
| *σ_C2_* | 0.0035 | Death rate of female calves, weeks 3 to weaning (per week) | [23] |
| *σ_C3_* | 0.00019 | Death rate of heifers from weaning to first calving (per week) | ^b^ |
| *σ_P1_* | 0.0056 | Initial culling rate of cows in parity 1 (per week) | [23] |
| *σ_P2_* | 0.0051 | Initial culling rate of cows in parity 2 (per week) | [23] |
| *σ_P3_* | 0.0066 | Initial culling rate of cows in parity 3 (per week) | [23] |
| *σ_P4_* | 0.0066 | Initial culling rate of cows in parity 4 (per week) | [23] |
| *σ_P5_* | 0.0184 | Initial culling rate of cows in parity 5 and above (per week) | [23] |
| *w* | 10 | Weaning age (weeks) | [23] |
| *y* | 52 | Age when entering the young heifer group (weeks) | [23] |
| *h* | 91 | Age at first artificial insemination (weeks) | ^a^ |
| *cal* | 130 | Age at first calving (weeks) | ^a, b^ |
| *cci* | 56.3 | Calving-to-calving interval (weeks) | ^a, b^ |
| *b* | 5 | Quantity of colostrum fed to calves (L/day for 3 days) | ^b^ |
| *d* | 7 | Quantity of milk fed to calves after 3 days (L/day/calf) | ^b^ |
| *prop* | 0.85 | Proportion of lactating cows | ^a^ |
| *ɛ* | 25 | Quantity of milk or colostrum produced (L/day/cow) | ^a^ |
| *f_1_* | 0.5 | Quantity of faeces produced by a non-weaned calf (kg/day) | ^b^ |
| *f_2_* | 5.5 | Quantity of faeces produced by a weaned calf (kg/day) | ^b^ |
| *f_Y_* | 10 | Quantity of faeces produced by a heifer (kg/day) | ^b^ |
| *f_A_* | 30 | Quantity of faeces produced by a cow (kg/day) | ^b^ |
| *graz* | [14-46] | Grazing period (1 being the first week of the year) | ^b^ |
| *K_c_* | 80 | Threshold number of cows in the herd |  |

^a^ Agricultural statistics

^b^ Experts’ opinions

**Model parameters for processes related to infection dynamics.**

| **Parameters** | **Values** | **Definition** | **Source** |
| --- | --- | --- | --- |
| *p_X_* |  | Probability of in utero transmission for a cow in health state X | [23] |
|  | *p_Il_* = 0.149 | X = latently infected (I_L_) |  |
|  | *p_Im_* = 0.149 | X = infected and moderately infectious (I_M_) |  |
|  | *p_Ih_* = 0.65 | X = infected and highly infectious (I_H_) |  |
| *u* | 52 | Maximum age in the susceptible compartment (weeks) | [23] |
| *h* | 0.1 | Susceptibility follows an exponential decrease exp(-h*age) | [23] |
| *v_X_* |  | Mean time spent in health state X (weeks) | [23] |
|  | v_It_ = 25 | X = latently infected (I_T_) |  |
|  | *v_Il_* = 52 | X = latently infected (I_L_) |  |
|  | *v_Im_* = 104 | X = infected and moderately infectious (I_M_) |  |
|  | *v_Ih_* = 26 | X = infected and highly infectious (I_H_) | ^a^ |
| *sh_X_* |  | Probability of shedding in colostrum or milk for cow in health state X | [23] |
|  | *sh_Il_* = 0 | X = latently infected (L) |  |
|  | *sh_Im_* = 0.4 | X = infected and moderately infectious (I_M_) |  |
|  | *sh_Ih_* = 0.9 | X = infected and highly infectious (I_H_) |  |
| *α* | 10^6^ | Map infectious dose | [23] |
| *β_l_* | 5 × 10^-4^× 7 | Transmission rate if ingestion of an infectious dose (per week) | ^b^ |
| *β_c_* | 5 × 10^-5^× 7 | Transmission rate if one infectious dose is present in the local environment (per week) | [23] |
| *β_g_* | 9.5 × 10^-7^× 7 | Transmission rate if one infectious dose is present in the general environment (per week) | [23] |
| *β_o_* | 5 × 10^-6^ × 7 | Transmission rate if one infectious dose is present on pasture (per week) | ^b^ |
| *g_X_* |  | Decreased rate of milk production for a cow in health state X (per week) | [23] |
|  | *g_Il_* = 0.08 | X = latently infected (I_L_) |  |
|  | *g_Im_* = 0.11 | X = infected and moderately infectious (I_M_) |  |
|  | g_Ih_ = 0.25 | X = infected and highly infectious (I_H_) |  |
| *µ_k_* |  | Removal rate of Map from environments | [23] |
|  | *µ_h_* = 0.4 | Housing environments |  |
|  | *µ_p_* = 0.07 | Pasture environments |  |

^a^ Experts’ opinions

^b^ Parameter values are assumed
