# Supplementary figures and images for: Control measures to prevent the increase of paratuberculosis prevalence in dairy cattle herds: an individual-based modelling approach
Source: Vet Res. 2018 Jul 13;49:60. doi: 10.1186/s13567-018-0557-3 (PMC6044053; doi:10.1186/s13567-018-0557-3)

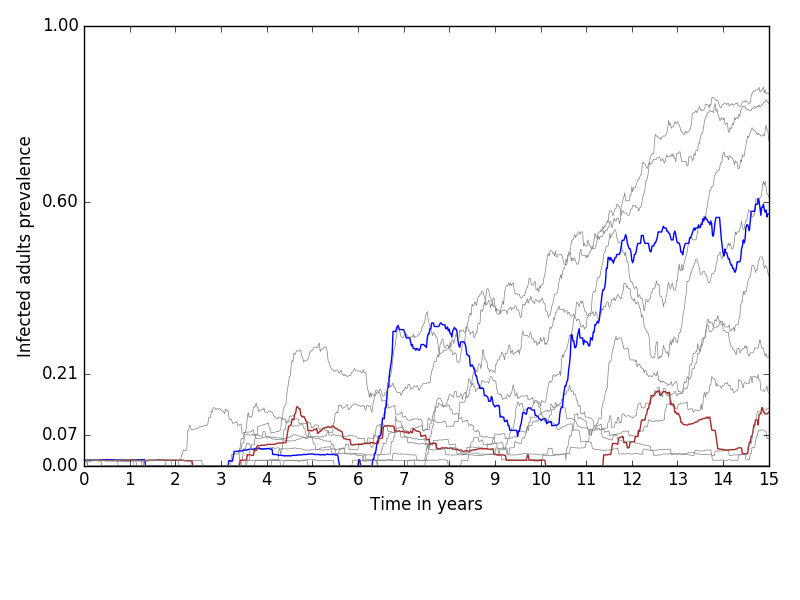

Supplement: Supplementary file 2 — Additional file 2. Sample of 11 trajectories of infected adult prevalence obtained with the reference scenario. Reference scenario was defined as the introduction of an infected heifer (IM) in a naïve herd and no control implementation. Each trajectory represents the variation of infected adult prevalence over time since Map introduction. The sample of 11 trajectories has been extracted from the 5000 trajectories used to build initial conditions. Two trajectories (blue and brown) have been highlighted to illustrate model stochasticity and the absence of an early epidemic phase followed by a steady-state prevalence on the contrary to what is classically encountered in epidemiology. [file 13567_2018_557_MOESM2_ESM.png]

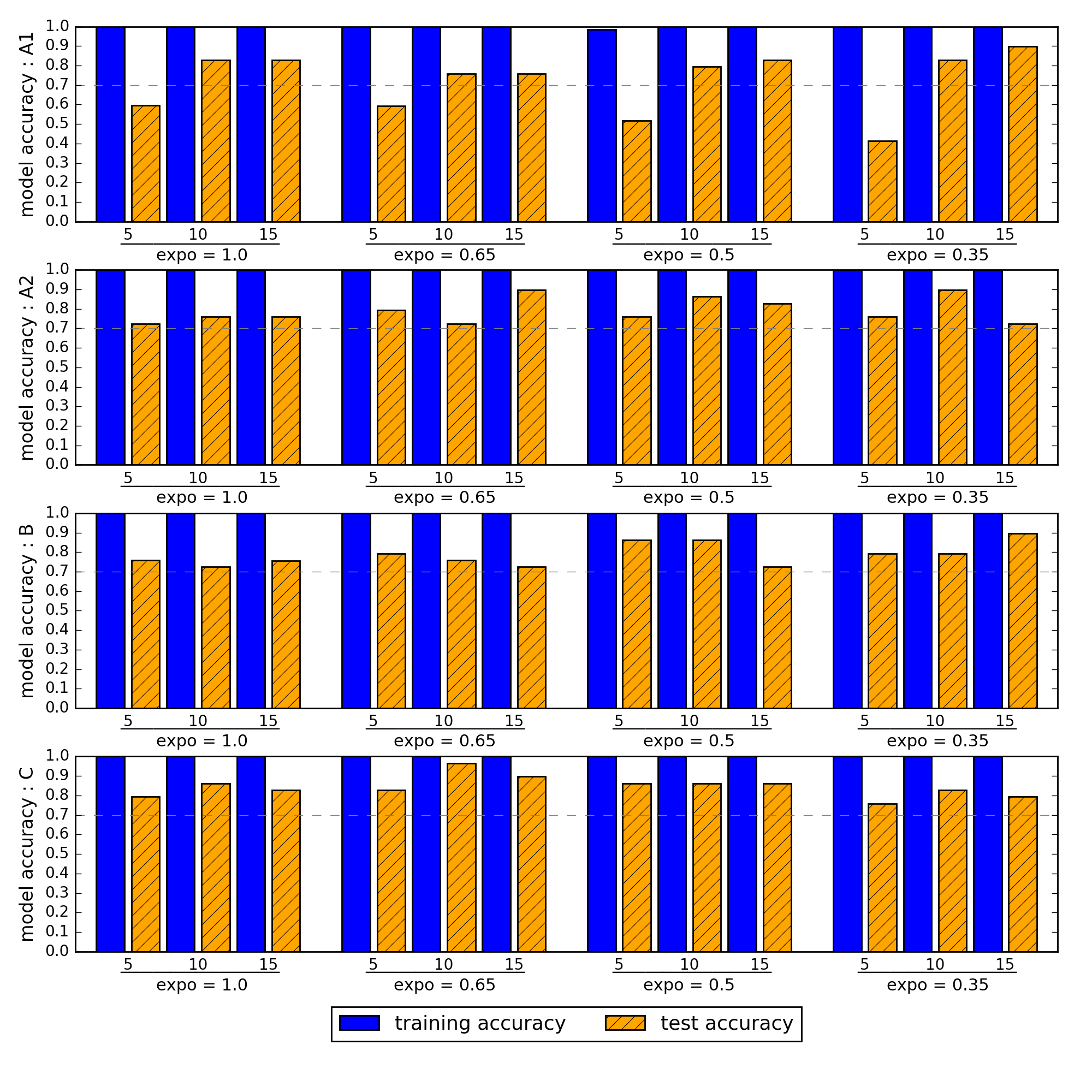

Supplement: Supplementary file 3 — Additional file 3. Precision of predictive statistical models built with the Random Forest Classifier method. All combinations of initial herd status (early low adult prevalence: A1; low adult prevalence: A2; moderate adult prevalence: B; high adult prevalence: C), year (5, 10, and 15 years), and calf exposure (no reduction: expo = 1; and 3 levels of reduction: expo = 0.65, 0.5, and 0.35) are shown. Accuracies are considered as good enough when above 70%. [file 13567_2018_557_MOESM3_ESM.png]

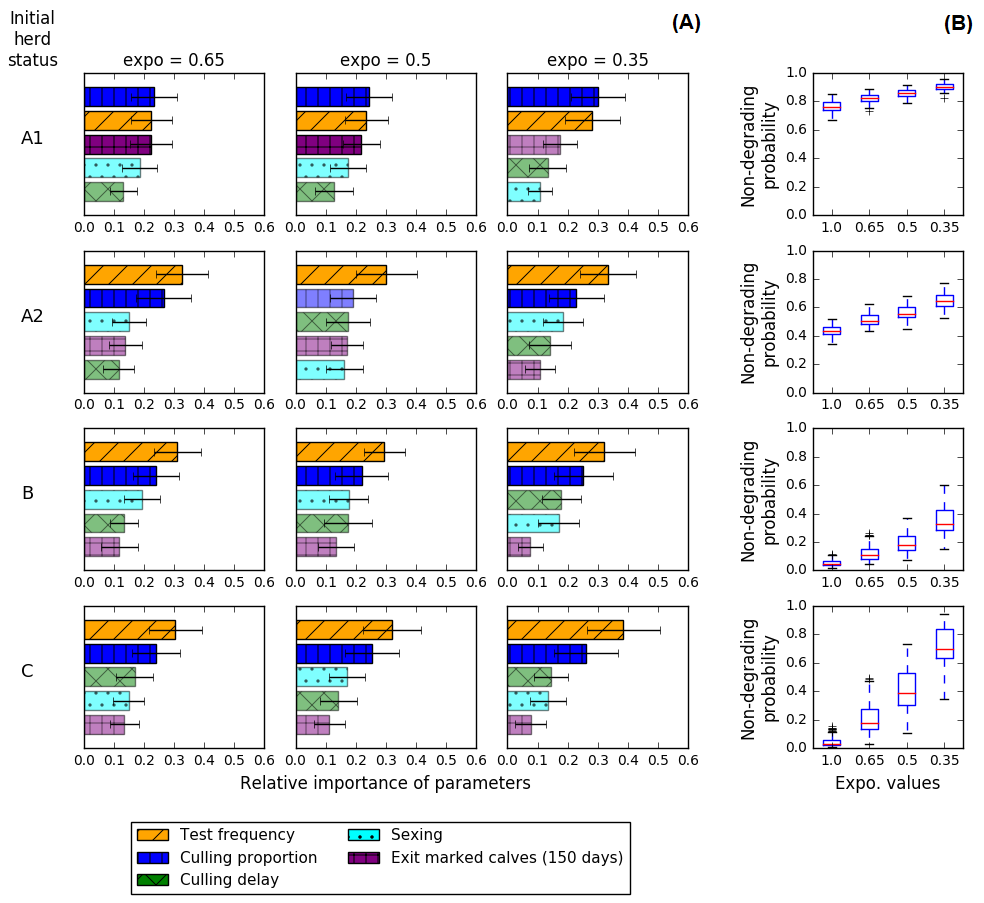

Supplement: Supplementary file 4 — Additional file 4. Influence of control measure modalities on the probability of non-degrading herd status over a 10-year period. (Panel A) Relative importance of each test-and-cull parameter linked to the predictive statistical model built with the Random Forest Classifier method, and (panel B) associated probability of non-degrading herd status over a 10-year period and according to initial herd status (A2, B, and C, in lines) and reduction of calf exposure (expo = 0.65, 0.5, and 0.35, in columns). [file 13567_2018_557_MOESM4_ESM.png]

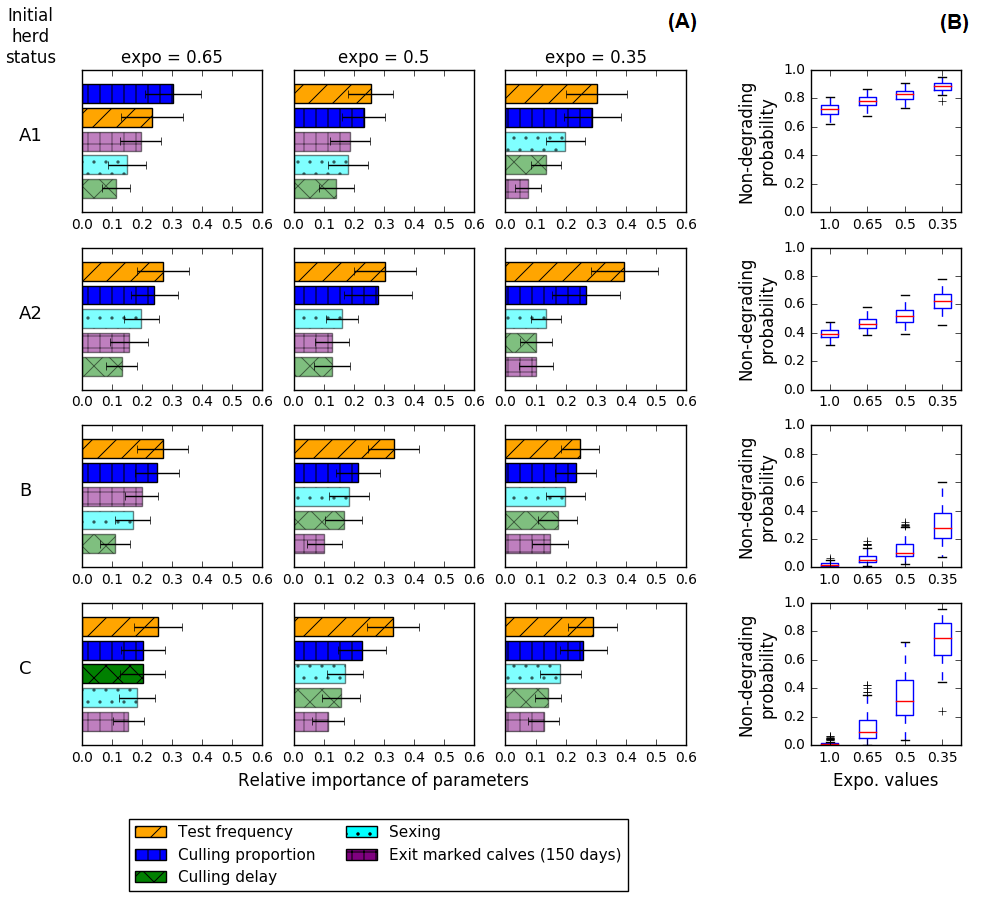

Supplement: Supplementary file 5 — Additional file 5. Influence of control measure modalities on the probability of non-degrading herd status over a 15-year period. (Panel A) Relative importance of each test-and-cull parameter linked to the predictive statistical model built with the Random Forest Classifier method, and (panel B) associated probability of non-degrading herd status over a 15-year period and according to initial herd status (A2, B, and C, in lines) and reduction of calf exposure (expo = 0.65, 0.5, and 0.35, in columns). [file 13567_2018_557_MOESM5_ESM.png]
